# Kinetic Analysis of the Uptake and Release of Fluorescein by Metal-Organic Framework Nanoparticles

**DOI:** 10.3390/ma10020216

**Published:** 2017-02-22

**Authors:** Tobias Preiß, Andreas Zimpel, Stefan Wuttke, Joachim O. Rädler

**Affiliations:** 1 Faculty of Physics and Center for NanoScience (CeNS), LMU Munich, Geschwister-Scholl-Platz 1, 80539 Munich, Germany; Tobias.Preiss@physik.lmu.de; 2 Department of Chemistry and Center for NanoScience (CeNS), LMU Munich, Butenandtstraße 11 (E), 81377 Munich, Germany; andreas.zimpel@cup.uni-muenchen.de

**Keywords:** metal organic framework, nanoparticle, loading and release

## Abstract

Metal-organic framework nanoparticles (MOF NPs) are promising guest-host materials with applications in separation, storage, catalysis, and drug delivery. However, on- and off-loading of guest molecules by porous MOF nanostructures are still poorly understood. Here we study uptake and release of fluorescein by two representative MOF NPs, MIL-100(Fe) and MIL-101(Cr). Suspensions of these MOF NPs exhibit well-defined size distributions and crystallinity, as verified by electron microscopy, dynamic light scattering, and X-ray diffraction. Using absorbance spectroscopy the equilibrium dissociation constants and maximum numbers of adsorbed fluorescein molecules per NP were determined. Time-resolved fluorescence studies reveal that rates of release and loading are pH dependent. The kinetics observed are compared to theoretical estimates that account for bulk diffusion into NPs, and retarded internal diffusion and adsorption rates. Our study shows that, rather than being simple volumetric carriers, MOF-NPs are dominated by internal surface properties. The findings will help to optimize payload levels and develop release strategies that exploit varying pH for drug delivery.

## 1. Introduction

The widespread use of porous materials in the field of separation, storage, and catalytic process technologies requires a thorough understanding of the adsorption and desorption of guest molecules within the porous structure. In this context, metal-organic frameworks (MOFs) are an interesting class of materials, as they are crystalline and, hence, possess a regular porous structure [1,2,3,4]. In MOFs, inorganic metal nodes connected by organic linkers create a diverse, but well-defined, chemical environment, which allows specific interactions with guest molecules. As a matter of fact, MOFs exhibit some of the highest porosities (1000 to 7000 m^2^/g) of all known porous solids, with pore sizes in the range of 0.3 to 6 nm [5]. Their high porosities and, in particular, the combination of high surface area with tunable pore size render MOFs ideal for applications in gas storage and separation [6,7], catalysis [8,9,10], sensing [11,12], electronics [13], drug delivery [14,15,16,17,18], and X-ray analysis of the structures of guest molecules within the MOF scaffold [4,19].

Recently, several reports have pointed to the general applicability of MOF nanoparticles (MOF-NPs) for drug delivery, as they have high loading capacities and are functionalizable, and certain structures have been shown to be biocompatible (e.g., MIL-100(Fe); MIL stands for Materials of Institute Lavoisier) [11,15,16,18,20,21,22,23,24,25]. MOF-NPs have been loaded with various drugs, including cisplatin [26], 5-fluorouracil [27], ibuprofen [28], doxorubicin, and cidofovir [29]. Both MIL-100(Fe) and MIL-101(Cr) represent good model materials for drug delivery, due to their large pores (diameters of 25–29 Å for MIL-100 and 29–34 Å for MIL-101) and window sizes (diameters of 5–9 Å for MIL-100 and 12–17 Å for MIL-101) [24,30,31]. MIL-100 and 101 show high chemical stability and typically large BET surface areas of up to 6000 m^2^/g for the bulk material (2000–4000 m^2^/g as nanoparticles) [32,33,34,35]. Indeed, in many respects, MIL-100(Fe) NPs are the most promising MOF-based vehicles available for drug delivery [24,29,36].

The ability of NPs made of solid materials to load and then specifically release drug molecules within the human body has been at the forefront of biomedical nanotechnology for more than a decade [37,38,39,40,41,42,43]. Yet studies on the loading and release kinetics of drugs in porous nanocarriers are very rare, even for established systems based on polymer, silica, or liposome particles [38,44,45,46]. One basic question that remains open is how pore size affects uptake and offloading. It is known that, within porous materials, diffusion coefficients are reduced by a factor 10^4^, as transport becomes an effectively 1D diffusion process [47]. Furthermore, the affinity of the cargo molecules for the internal surface of the porous material (host-guest interaction) is likely to play an important role in determining the kinetics of transport, as well as the loading capacity [48]. Within the diffusion-immobilization model it is conceived that molecules undergo repeated cycles of absorption, desorption, and brief spells of free diffusion before an equilibrium situation is reached [49,50]. In addition, the conditions will change during the course of in vivo delivery. Affinity is likely to depend on the pH value of the environment, owing to the influence of pH on the charge of both cargo and MOF. As the pH varies within the human body, release kinetics will vary with local acidity. With the use of MOF-NPs as reliable and tunable drug carrier systems in mind, characterization of host-guest interaction and release is essential for optimized dosing.

In this work, we study the loading and release kinetics of MIL-100(Fe) and MIL-101(Cr). Our goal is to elucidate—on the basis of these representative MOF-NPs—the mechanisms and limiting factors that drive and constrain, respectively, molecular transport in and out of porous NPs, and compare these results with theoretical estimates. To this end, we characterize the MOF-NPs using transmission electron microscopy (TEM), dynamic light scattering (DLS), and X-ray diffraction (XRD), and measure the uptake of fluorescein via fluorescence spectroscopy at various pH values. Fluorescein was used because its size is comparable to common drug molecules and it is possible to quantify it by fluorescence and absorption. We find that MIL-100(Fe) and MIL-101(Cr) NPs have well-defined size distributions and crystallinity, and remain crystalline in buffer. DLS and zeta-potential measurements show that NP agglomeration is strongly pH dependent. By performing titration studies we determined the dissociation constants for fluorescein (disodium salt) and find that the NPs have a high payload capacity, which is compatible with the internal area estimated from BET measurements. Kinetic fluorescence studies show fast loading kinetics with high affinity in (unbuffered) distilled water (at low pH) and slower loading kinetics (i.e., lower affinity) at high pH (7.4–8.4), while release shows the converse behavior: high affinity and slow release at low pH (and in water). We show that loading and release kinetics can be theoretically described by diffusion to target, followed by restricted internal diffusion and equilibrium binding to the internal surface (physisorption). These findings demonstrate that physicochemical studies of MOF-NP loading enable rational, predictive design of release scenarios, particularly with regard to varying pH conditions.

## 2. Results and Discussion

In all following experiments, we study MOF-NPs of types MIL-100(Fe) and MIL-101(Cr), which were synthesized as described in Wuttke et al. [51] Prior to the loading and release studies, we characterized the size distribution of the MOF-NPs using DLS, FCS, and TEM [52], their major structural features by XRD, and their porosities by measuring nitrogen adsorption and deriving sorption isotherms to confirm the expected regular porosity of MOF-NPs.

**Figure 1:**
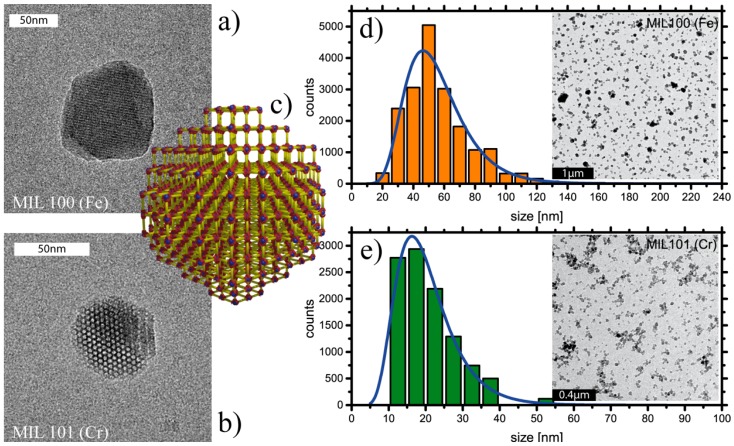
(**a**,**b**) TEM images of the two MOP-NP types used here show mesoporous structure and shape; (**c**) Simplified depiction of the crystalline structure with hollow pores taking up most of the volume; yellow rods with red ends: organic linker, blue dots: metal centers; (**d**,**e**) Size histogram of MOF-NPs based on particle analysis of electron micrographs yields a typical size for MIL-100(Fe) of 53 nm and for MIL-101(Cr) of 19 nm.

TEM images of MIL-100(Fe) and MIL-101(Cr) NPs reveal particles with an approximately spherical shape (Figure 1). Moreover, the TEM images indicate high crystallinity of the particles, as evidenced by the presence of electron diffraction fringes. We analyzed the size distribution based on different TEM images of MOF-NPs (see Supplementary material) [52]. Over 10,000 particles were examined for their projected size, assuming sphericity, and employing image analysis for the separation of closely adjacent particles (for details, see Supplementary material). The size histograms of both MOF-NPs reveal a slightly polydisperse (σ > 5% [53,54]) distribution (Figure 1d,e). MIL-100(Fe) NPs have a mean diameter of 52.4 nm (σ = 32%, FWHM 30.9–69.5 nm), whereas MIL-101(Cr) NPs have a mean size of 18.9 nm (σ = 35%, FWHM 10.3–25.7 nm). We utilized this information to estimate numbers of NPs per volume given an estimate of NP mass based on the crystallographic mass densities [30,31]. For MIL-100(Fe) NPs we used a mean radius of *r_MIL_*_–100_ = 26.5 nm and a mass density of ρ*_MIL–_*_100_ = 0.98 g/mL [30]. We obtained a mean mass per NP of *m_MIL_*_–100_ = 76 × 10^–18^ g and, thus, a number density of *N_MIL_*_–100_ = 1.31 × 10^13^ NPs per mg (for details, see SI). This corresponds to an NP number concentration of *n_MIL_*_–100_ = 21.7 pmol. Using the corresponding values *r_MIL_*_–101_ = 9.45 nm and ρ*_MIL_*_–101_ = 0.62 g/mL [31], we derived a mean particle mass of *m_MIL_*_–101_ = 2.2 × 10^–18^ g and thus *N_MIL_*_–101_ = 4.56 × 10^14^ particles per milligram (*n_MIL_*_–100_ = 760 pmol). These values were subsequently used to calculate molecular loading per NP.

To complement the information derived from 2D projections of NPs imaged by TEM, DLS-based analysis of MOF-NPs in solution (see Supplementary material) provided information on their diffusive behavior and, hence, on the hydrodynamic radius of the NPs. In accordance with results reported in the literature [51], MIL-100(Fe), and MIL-101(Cr) NPs have hydrodynamic diameters of about 124 nm and 69 (±19) nm respectively. Comparison of these observations with the TEM size distribution results suggests that the NPs tend to form small agglomerations in unbuffered water. XRD measurements (see Supplementary material) confirm the crystallinity of the MOF-NPs observed in the TEM images [30,31].

In order to verify the stability of the particles over the time scales employed for loading and release, XRD measurements were performed on NPs that had been incubated in buffer for 1 h. The results (see Supplementary material) show no significant change in the diffraction pattern, indicating that there is no structural change in the NPs.

On examining the size distributions of the NPs in the presence of various concentrations of fluorescein with DLS, we noted that the size of MIL-100(Fe) NPs increases slightly with increasing concentrations of fluorescein. This indicates that NPs tend to aggregate under varying fluorescein concentrations. One possible explanation is the alkalinity of fluorescein disodium salt, which will lead to concentration-dependent changes in pH. Electrostatic interactions between charged molecules or “crosslinking” of MOF NPs by fluorescein molecules, as has been found for, e.g., doxorubicin [55] might also contribute to this effect. In order to examine these possibilities more closely, we performed DLS and concurrent zeta-potential experiments on suspensions of MOF-NPs in water. The pH was increased incrementally in steps of 0.5 units (the initial suspension of MOFs in water has a pH of 2) by adding NaOH (see Supplementary material), allowing us to study the pH dependency of effective particle size in a well-defined system. DLS analysis yields an initial size of about 200 nm for MIL-100(Fe) and about 50 nm for MIL-101(Cr). With increasing alkalinity the zeta-potential drops, and below a value of about ±25 mV particles tend to agglomerate. This finding is in agreement with the previous observation that a zeta-potential of greater than 25 mV (absolute value) is required for NPs to be stabilized by electrostatic repulsion [39,56]. In the case of MIL-100(Fe) NPs, the zeta-potential drops to negative values at pH values higher than 5.5. This leads to newly-emerging repulsion forces, so that agglomerates tend to separate again. The strong dependence of particle size and zeta-potential on the pH of the local environment is taken into account in our theoretical model (see below), but this could be avoided by appropriate coating of the MOF-NPs [36,51,57].

**Figure 2:**
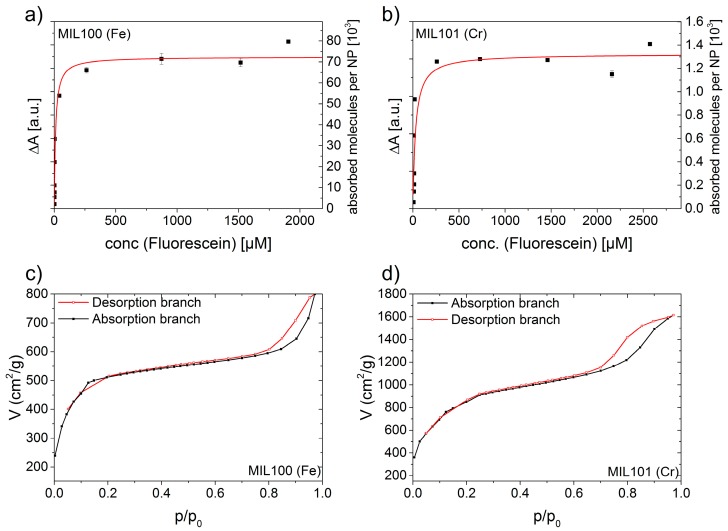
(**a**,**b**) Amounts of fluorescein loaded into MOF NP (obtained from the difference in absorption between the starting fluorescein solution and the supernatant recovered after loading) as a function of external fluorescein concentration fit to Langmuir-type curves. The calculated dissociation constants and maximum payload capacities per mg of NPs are: $K_{D}^{MIL100}$ = 11 μM, $K_{D}^{MIL101}$ = 136 μM, $P_{\max}^{MIL100}$ = 649.4 μg, $P_{\max}^{MIL101}$ = 413.5 μg (**c**,**d**) Measurements of nitrogen gas absorption by the MOF-NPs. The BET surface area obtained for MIL-100(Fe) NPs is 2004 m^2^/g and for MIL-101(Cr) is 3205 m^2^/g. Taking both into account yields a mean area occupied by one fluorescein molecule of 2 nm^2^ for MIL-100(Fe) and 5 nm^2^ for MIL-101(Cr).

We then turned to the loading behavior, and determined the dissociation constants and the maximum capacities of MOF-NPs for uptake of fluorescein. For this purpose NP suspensions that had been incubated for a certain time (24 h) in fluorescein solutions of different concentrations were centrifuged, and the fluorescein remaining in the supernatant was quantified by UV-VIS absorption using a calibration curve based on a fluorescein dilution series (see Supplementary material). The difference in absorbance between the starting solutions and the supernatants recovered after centrifugal removal of both types of MOF-NPs is shown in Figure 2a,b (for details, see Supplementary material). We used initial fluorescein concentrations of between 20 μg/mL and 1500 μg/mL. Each data point represents the average of three independently prepared and measured samples. The data were fitted to a Langmuir-type sorption function:

(1) $$P\left(c\right)=\frac{P_{\max}\cdot c}{c+K_{D}}$$

Here, *c* is the concentration of fluorescein, *P*_max_ is the saturation value of adsorbed fluorescein, and *K*_D_ is the dissociation constant (i.e., the concentration at which half of the maximal possible fluorescein is adsorbed). Both MIL-100(Fe) with $K_{D}^{MIL-100}$ = 4.4 μg/mL = 11 μM and MIL-101(Cr) with $K_{D}^{MIL-101}$ = 11.7 μg/mL =36 μM were found to have low dissociation constants, both compared to that of doxorubicin bound to MIL-100(Fe) as determined by Anand et al. (91 μM) and in light of its high maximal capacity for adsorbed fluorescein ($P_{\max}^{MIL-100}$ = 649.4 μg =1.6 μmol and $P_{\max}^{MIL-101}$ = 413.5 μg =1.0 μmol) [55]. We convert the adsorbed mass of fluorescein per mass unit of nanomaterial into a molar ratio (number of adsorbed fluorescein molecules per NP) using the molar mass of the NPs obtained from TEM analysis and $M_{\mathrm{FC}}$ = 412.3 g/mol for fluorescein disodium (see Supplementary material for further details). The calculated number of adsorbed fluorescein molecules per single NP is shown in Figure 2 (right axis). The large numbers (on the order of 10^3^ to >10^4^) indicate the high payload capacity of the MOF NPs. Note that these loading capacities correspond to a weight payload ratio (load weight/carrier weight) of 41% for MIL-101(Cr) and 65% for MIL-100(Fe). The latter is in good agreement with published data for other guest molecules [28,29,55]. We also constructed N_2_ isotherms (Figure 2) for comparison of the amount of loaded fluorescein molecules with the accessible internal surface area of the MOF-NPs. The corresponding BET surface area is estimated to be $S_{\mathrm{BET}}$ = 2004 m^2^/g for MIL-100(Fe) and $S_{\mathrm{BET}}$ = 3205 m^2^/g for MIL-101(Cr). By combining the maximum payload capacity per mg NPs with the BET surface results, we calculate the area occupied by one fluorescein molecule (*A*_FC_) for both types of MOF-NPs: $A_{\mathrm{FC}}^{MIL-100}=S_{\mathrm{BET}}^{MIL-100}/P_{\max}^{MIL-100}\cdot 1\;\mathrm{mg}=2\;{\mathrm{nm}}^{2}$ and $A_{\mathrm{FC}}^{MIL-101}=S_{\mathrm{BET}}^{MIL-101}/\;P_{\max}^{MIL-101}\;\cdot 1\;\mathrm{mg}=5\;{\mathrm{nm}}^{2}$. For comparison, a single fluorescein molecule has an approximate projection area of about 1.1 nm^2^ (see Supplementary material). Hence, we can assume that the internal surface of both MOF-NPs is densely packed with fluorescein molecules.

**Figure 3:**
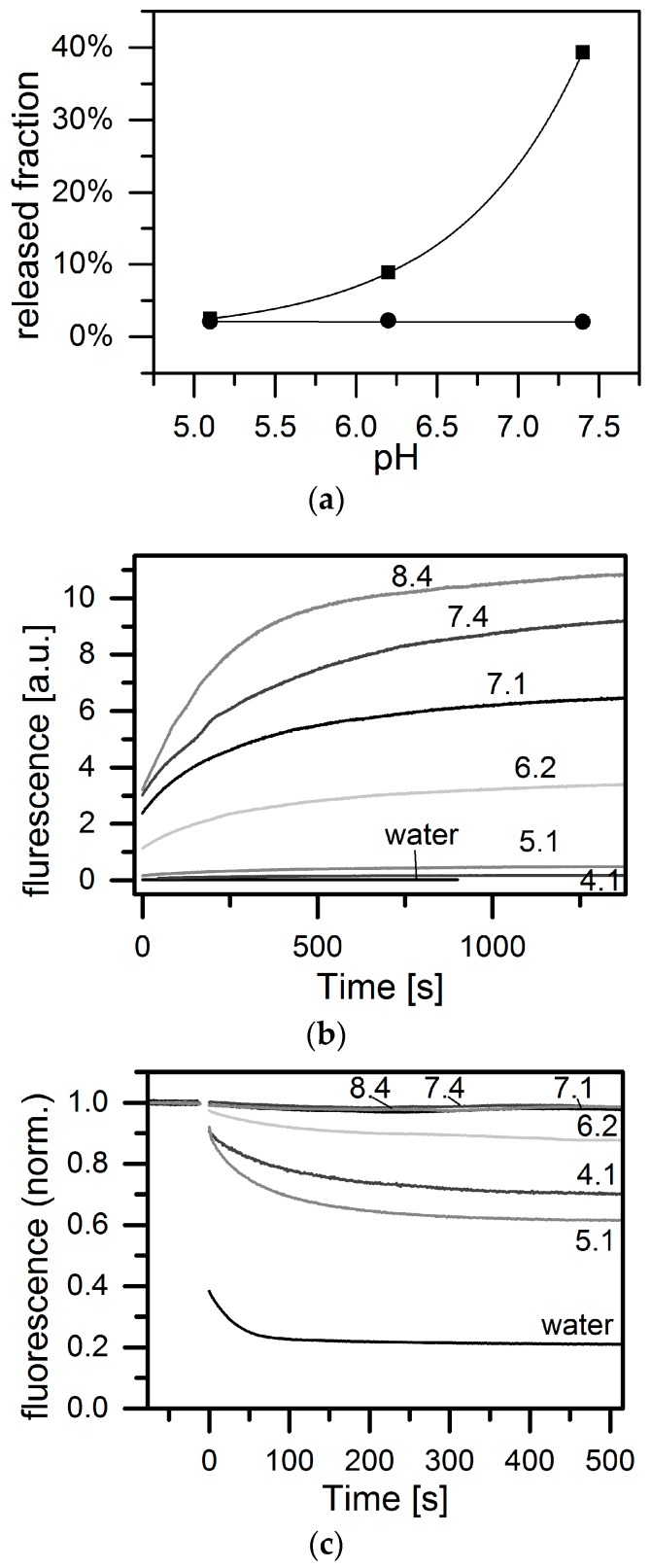
(**a**) Fraction of fluorescein released from prefilled MOF-NPs after 90 min in buffer (HBG) at different pH, determined by absorption measurements of supernatant containing free fluorescein. While MIL-101(Cr) (circles) shows almost no (<3%) release of fluorescein at any pH tested, for MIL-100(Fe) (squares) we observed a significant increase in release with rising pH (exponential fit to guide the eye); (**b**) Fluorescence quenching over the time course of release. MIL-100(Fe) nanoparticles filled with fluorescein were suspended in HBG buffered at different pHs. In water there is no increase in fluorescence intensity over time, indicating that there is no release; (**c**) Fluorescence quenching in the time course of loading. Fluorescein solution in HBG buffer at different pH and in water before and after addition of MIL-100(Fe) nanoparticles. In water the loading is the fastest and most efficient. In HBG at pH 4.1 to 6.2 it is slower and less efficient while at pH 7.1 to 8.4 no loading is observed at all.

We next addressed the questions of whether the entire payload can be released by reducing the external concentration of fluorescein, and whether this occurs on a reasonable timescale. To investigate offloading we measure the amounts of fluorescein molecules released by both types of MOF-NPs. To this end, MOF-NPs filled with fluorescein were resuspended in HBG buffer (20 mM HEPES + 5% glucose) at the physiologically relevant pH values of 5.1 (late endosome), 6.2 (early endosome) and 7.4 (blood) [58]. After 90 min, particles were removed by centrifugation and the absorbance of the supernatant was measured via UV-VIS (Figure 3). As future pharmaceutical applications will need cell culture experiments, we decided to use HBG as the environment for our experiments to have a cell culture approved buffer. As a reference for 100% release the absorbance of fluorescein solutions prepared in HBG at the same pH and concentration as the test solutions were used. In the case of MIL-101(Cr), almost no release (<3%) is observed within 90 min, while for MIL-100(Fe) the amount of released fluorescein increased with rising pH from below 3% at pH 5.1 to about 40% at pH 7.4. Thus, it appears that fluorescein binding to MIL-101(Cr) is essentially irreversible under our conditions, or at least exhibits very extremely long off-times.

The pH-dependent release from MIL-100(Fe) deserves further attention. We used time-resolved fluorescence measurements to determine the kinetics of MIL-100(Fe) loading and release, making use of the fluorescence quenching effect observed when fluorescein molecules bind to the porous scaffold of MIL-100(Fe) NPs. Since MIL-101(Cr) does not exhibit this quenching effect, this assay cannot be used on these NPs. Prefilled MIL-100(Fe) NPs were centrifuged and the remaining supernatant was removed. Then the fluorescein-loaded MIL-100(Fe) NPs were re-suspended in HBG buffer at various pH values (pH = 4.1, 5.1, 6.2, 7.1, 7.4, and 8.4). Subsequently, the fluorescence signal originating from the fluorescein released from the MIL-100(Fe) NPs was recorded over time (see Figure 3b). The fluorescence signal at late time points increases with increasing pH, although the total amount of fluorescence released is more or less the same at all pHs tested, as can be seen when the fluorescence yield at the respective pH is taken into account. However, no rise in the fluorescence signal is seen in (unbuffered) water, indicating that no release occurs at all at the low pH of the suspension. When the fluorescence intensity after release into buffered medium was compared with that of the supernatant recovered after loading, it emerged that almost all of the fluorescein bound by the NPs is released again. When considering the release time traces in buffer with respect to the rates of fluorescein release, it is useful to normalize the data to the final fluorescence signal as shown in SI. Apart from the measurement at pH 8.4, all release curves end up stacked on top of each other, indicating that the temporal characteristics of cargo release are the same for all pH values.

These results require a detailed look at the on-loading kinetic. Loading was monitored by measuring the fluorescence of a 2 mL aliquot of dilute (0.1 μM) fluorescein solution from the moment a small amount (10 μg) of MIL-100(Fe) NPs was mixed into the solution. This was done for fluorescein dissolved in water and in HBG buffered at pH values of 4.1, 5.1, 6.2, 7.1, 7.4, and 8.4. The fluorescence of the solution was measured over time and normalized with respect to the fluorescence signal of the respective starting fluorescein solution without MOF-NPs (Figure 3c). This signal shows a significant decrease over time, which is interpreted as reflecting the decreasing amount of fluorescein remaining in solution due to uptake (and fluorescence quenching) by the MOF-NPs. Inspection of the normalized fluorescence signal after >400 s of loading time reveals a clear trend: In the case of distilled water (MilliQ), the fluorescence drops to ≈20% of the signal prior to NP addition. The drop is less obvious when loading is carried out in buffer (at all tested pHs from 4.1 to 8.4). In the latter case, however, a strong pH dependence is found: the initial level of fluorescence declines by about 35% at pH 4.1, the corresponding value at pH 6.1 is 14%, and no detectable change in fluorescence is observed at pH > 7. We, therefore, assume there is no uptake into the NPs under alkaline conditions, and no quenching of fluorescein. Thus, we find a clear dependence of the loading rate upon the pH, as revealed by the rate of decay of the fluorescence signal. To quantify this, we fitted an exponential decay to the data for the kinetics of loading (see Supplementary material). The resultant loading times are shown in Table 1. While loading takes place very rapidly in water, uptake rates in buffer fall with rising pH, and no loading can be quantified at pH 7.1 or higher. 

**Figure 4:**
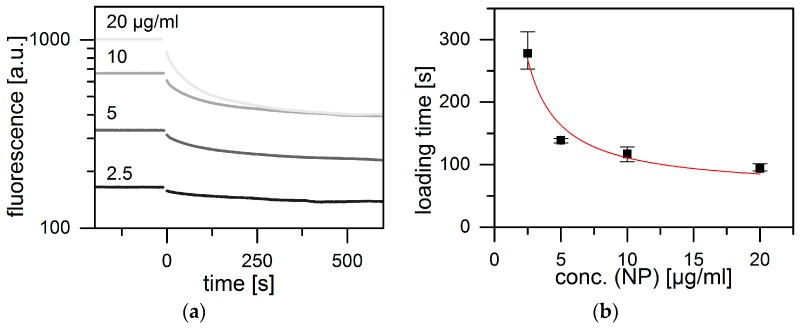
Fluorescence quenching in the time course of loading at various MIL-100(Fe) NP concentrations in HBG (pH 5.1) at fixed fluorescein to NP ratios. (**a**) Kinetics of the decay of fluorescein fluorescence after addition of NPs at time = 0. Time traces were fitted with single exponential decay; (**b**) The resulting loading times (in black) show a characteristic concentration dependency. This fits well with a model (red) involving a three-step process: free external diffusion, internal diffusion within the lattice and adsorption to the MOF network.

**Figure 5:**
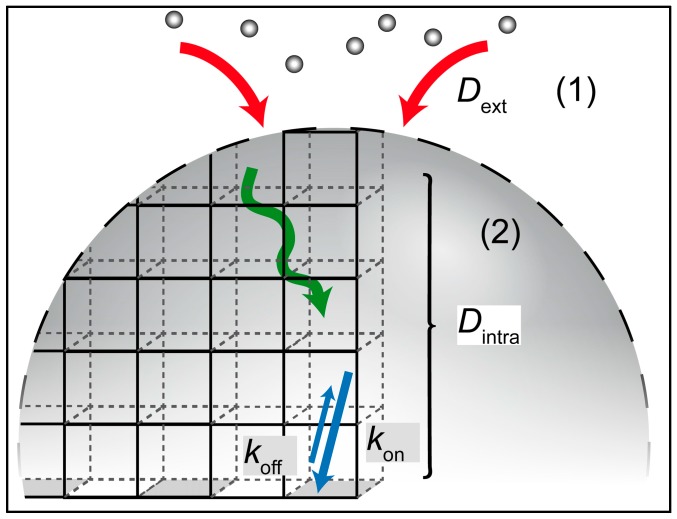
Illustration of the mass transport during loading: (1) external diffusion of fluorescein molecules towards the nanoparticles as described by the Adam and Delbrück model; (2) Concurrent intracrystalline diffusion and adsorption/desorption to/from the internal surface of the MOF NP (reaction-diffusion).

Next we asked whether the observed loading kinetics can be understood as a reaction-limited diffusion process. To this end, we studied the time course of the change in the fluorescein signal during uptake by MIL-100(Fe) at various NP concentrations but constant fluorescein/NP ratio. In this way, the average distance a fluorescein molecule has to diffuse before reaching the NP surface is varied. Experiments were carried out at constant pH of 5.1. The fluorescence time courses decay exponentially for all concentrations, as shown in Figure 4a. As before, we assume that fluorescein is quenched during adsorption to the internal MOF surface and, hence, that the fluorescence decay is a measure for the rate of loading. Data were fitted by single exponentials and the derived characteristic loading times were plotted as a function of NP concentration (Figure 4b). If the loading is dominated by diffusion of molecules from the bulk phase to the MOF surface, we can calculate the on-kinetics and compare the result to the data in Figure 4b. The expected time for diffusion to NP surfaces is estimated assuming that, for each NP, molecules are recruited from a spherical volume with a radius equal to half the average NP-NP distance. Diffusion of molecules in a spherical volume with radius *R* to a spherical absorber with radius *r*, in the center of that volume is described by the theory of Adam and Delbrück [59]. As further explicated in the SI we derive an estimate for the spherical radius *R* from the NP concentration. With this we obtain a typical diffusion-limited time for the capture of fluorescein (see Supplementary material):

$$\tau_{\mathrm{diff}}\left(c_{\mathrm{NP}}\right)\approx \frac{\pi \;r^{2}\;\rho }{18\;c_{\mathrm{NP}}\;D_{\mathrm{ex}}}$$

where *D*_ex_ is the external bulk diffusion coefficient of fluorescein, *r* the NP radius, ρ the NP mass density in $\mathrm{mg}/{\mathrm{cm}}^{3}$, and *c*_NP_ the NP concentration in mg/cm^3^. Hence, the external diffusion time is predicted to decay in proportion to *c*_NP_^−1^. The experimental loading times follow this prediction, as shown in Figure 4. The unbroken curve represents a fit to $A\cdot c_{NP}^{-1}+\tau_{0}$. The prefactor, *A*, is in good agreement with the time predicted assuming an effective density of ρ = 2 mg/cm^3^ for the MOF-NPs (see also Supplementary material). However, there remains a finite loading time offset, τ_0_ even at high NP concentrations, when diffusion time to the target becomes negligible. The latter offset time subsumes all internal processes that occur subsequently to diffusive transport to the NP, including internally hindered diffusion through the porous lattice, sorption to the internal surface and possibly surface rearrangements. A schematic representation of the molecular transport processes inside the mesoporous scaffold is depicted in Figure 5. If we consider a typical NP diameter to be of the order of 50 nm, then the corresponding internal diffusion constant (*D*_intra_ ≈ *r*^2^/*t*_0_) would be of the order of 10^−17^ m^2^/s and, hence, 10^7^ times smaller than the bulk diffusion constant (3.9 × 10^−10^ m^2^/s) measured for fluorescein in water (see also supplementary information). Hence, the observed offset time, τ_0_ ≈ 60 s, is surprisingly long. Possible explanations are the strong binding of fluorescein to the internal surface, slow relaxation processes take place in the adsorbed internal monolayer of fluorescein, or surface defects that lead to hindered diffusive entry) of fluorescein into the MOF NPs [60].

## 3. Materials and Methods

### 3.1. Equilibrium Measurements

Payload capacity was measured using a UV-VIS absorption spectrometer (NanoDrop 1000, Thermo Scientific, Waltham, UK). MOF-NPs (1 mg) in ethanol stock solution were centrifuged (45 min at 14,680 rpm, 20,238× *g*) to remove the supernatant ethanol. The pellet of MOF NPs was then dispersed in an aqueous dilution series of fluorescein sodium salt (Sigma-Aldrich, St. Louis, MO, USA) by vortexing and sonication (Bandelin Sonorex, Berlin, Germany), and incubated for 96 h under continuous agitation in a tube rotator. The suspensions were then centrifuged as before to obtain the supernatant fluorescein solution. The absorption spectra of the supernatant, as well as that of the original fluorescein solution, were measured and the area under the curve between 400 to 550 nm, hereinafter denoted as absorbance (see Supplementary material), was determined (OriginPro 9 64-bit, OriginLab, Wellesley Hills, MA, USA). This procedure was performed for a concentration series of fluorescein solutions ranging from 5 μg/mL to 1500 μg/mL. A straight line *A* = *m·c + t* was fitted to the integrated absorbance of the original fluorescein solution concentration series, where *A* is the measured absorbance and *c* the concentration of the original fluorescein solution (inset in Figure S2b).

To determine the amount of fluorescein released, 1 mg of MOF-NPs was first loaded with the compound by suspension in 1 mL of an aqueous solution (100 μg/mL) of fluorescein and incubated for 1 day on a rotary shaker at room temperature. Subsequently the nanoparticles were transferred into 1-mL aliquots of freshly-prepared HBG buffer at pH 5.1, 6.2, and 7.4 by centrifugation (15/45 min at 20,238× *g*), removal of the supernatant and resuspension in buffer. This was followed by 90-min incubation on the rotary shaker at room temperature. After final removal of the nanoparticles by centrifugation for 45 min as before, the absorption spectrum of the supernatant was measured. As a reference for 100% release, the absorption spectra of 100 μg/mL solutions of fluorescein in HBG buffered at pH 5.1, 6.2, and 7.4 were also obtained. The spectra were integrated over the range between 400 and 550 nm (OriginPro 9 64-bit) and the resulting absorbance of the released fluorescein solutions was compared with the reference absorbance at the same pH.

### 3.2. Kinetics of Loading/Release

Loading: For each measurement, a 2-mL aliquot of fluorescein solution (0.1 μg/mL ≈ 0.24 μmol), made up in water or HBG at pH 4.1, 5.1, 6.2, 7.1, 7.4, or 8.4, was filled into a polystyrene cuvette. The fluorescence signal (divided by the instrument’s lamp reference to correct for fluctuations in lamp brightness) emitted upon excitation at 492 nm (slit width, 3 nm) was recorded for at least 60 s in a Fluorolog 3 spectrometer (Horiba, Miyanohigashi, Japan) at 512 nm. Then 2 μL of MIL-100(Fe) suspension (=10 μg) in aqueous ethanol (5 mg/mL) was quickly pipetted into the cuvette and mixed, and the instrument cover was closed again (denoted as *t* = 0 s). The fluorescence signal was then monitored over the course of at least 500 s.

Release: For each measurement, 50-μg samples of NPs that had been incubated in 0.5 μg/mL fluorescein were recovered by centrifugation (for 15 min, as above), and the supernatant was discarded. The pellet was then re-suspended in 10 mL of water or HBG (buffered at one or another of the pH values mentioned above) by sonication (see above), and a 2-mL portion was rapidly transferred to a cuvette and the fluorescence signal was measured for at least 700 s as described above.

Sorption measurements (BET): Nitrogen sorption isotherms were measured at 77 K with a Quantachrome NOVA 4000e (Boynton Beach, FL, USA). Approximately 20 mg of nanoparticles was degassed at 150 °C in high vacuum for at least 12 h prior to measurement. Evaluation of the sorption data was carried out using ASiQwin™ software (Version 2.0, Quantachrome Instruments). BET surface areas were calculated with the linearized form of the BET equation. For all samples the correlation coefficient was higher than 0.999. Adsorption isotherms were used to calculate the pore size distribution by quenched-solid density functional theory (QSDFT, N_2_ at 77 K on carbon, cylindrical/spherical pores adsorption branch).

Transmission Electron Microscopy (TEM): For TEM analysis 10 μL aliquots of ethanolic MOF-NP suspension were dried on 300 mesh Formvar/carbon copper grids (Ted Pella, Redding, CA, USA). Pictures of MOF NPs on grids were obtained on a JEM 1011 (JEOL, Tokyo, Japan) at an acceleration voltage of 80 kV.

X-ray diffraction (XRD): For XRD measurements, approx. 1 mg of the powdered material was distributed homogeneously between two acetate foils (ultraphan) with a thickness of 0.014 mm and fixed in the sample holder. The samples were the measured with the transmission diffractometer system Stadi MP (STOE, Darmstadt, Germany) with Cu Kα1 radiation (λ = 1.54060 Å) and a Ge(111) single-crystal monochromator. Diffraction patterns were recorded with a solid-state strip detector MYTHEN 1K (DECTRIS, Baden-Daettwil, Switzerland) in omega-2-theta scan mode using a step size of 4.71° and a counting time of 80 s per step.

Dynamic light scattering (DLS) and zeta-potential measurements: DLS and zeta-potential measurements of the particles in dispersion (approx. 0.1 mg/mL) were carried out using a Malvern Zetasizer (Nano Series, Nano-ZS, Herrenberg, Germany). For measurements of the pH dependence of the zeta-potential, the instrument was equipped with a Malvern Multi-Purpose Titrator (MPT-2, Herrenberg, Germany). A 10-mL aqueous suspension of nanoparticles (0.1 mg/mL) was set to the starting pH with HCl (0.1 M) and titrated in steps of 0.5 pH units with NaOH (0.01 or 0.1 M, respectively) up to the final pH value.

## 4. Conclusions

In summary, we have studied the loading (release) of a model guest molecule (fluorescein) into (from) porous MOF-NPs. We found for both studied NP types, MIL-100(Fe) and MIL-101(Cr), that significant amounts of fluorescein can be adsorbed at room temperature. The measured loading capacities, in the range of >10³ molecules per NP, are compatible with the measured internal surface area available. The loading rate in the case of MIL-100(Fe) is found to be dependent on the pH and the solvent (water or HBG). Our studies show that optimal loading of fluorescein is achieved in MilliQ water, and no release from the NPs is detected in this case. Unlike loading, however, the pH dependence of payload release varies between the two types of NPs studied. Virtually no release from MIL-101(Cr) occurs at any of the pH values tested, whereas MIL-100(Fe) NPs release between 3% (at pH 5.1) and about 40% (at pH 7.4) of their adsorbed fluorescein. These findings suggest that electrostatic interactions possibly contribute to the confinement of the guest molecules inside the MOF pores. Considering the versatile MOF chemistry, as well as the different ways how to functionalize a MOF scaffold encompass a controlling of the MOF host-guest interactions.

Thus, MOF nanocarriers are good candidates for drug delivery and other applications where a high payload is desirable. In addition, MIL-100(Fe) shows release characteristics that can be tuned via pH. The latter result demonstrates that controlled release from MOF-NPs can be detected when loading and offloading of payload molecules by these nanocarriers are characterized. This information is vital for clinical applications as a possible drug delivery system. However, only a small number of relevant drugs exhibit optical fluorescence or optical adsorption changes that can be exploited for time-resolved release studies. Thus, there remains a need for alternative characterization methods to assess loading and release behavior, and to optimize MOF nanocarriers for regulated drug delivery using refined chemical functionalization.

## Figures and Tables

**Table 1:** Results obtained from single exponential decay fitting of loading kinetics in water and HBG buffer at pH 4.1 to 8.4. While the loading process is very fast in water, in buffers with defined pH values rates of loading fall with rising pH, and no loading is detectable at pH 7.1 or higher.

pH	Rates of Decay (10^–3^·s^–1^) (from Exponential Fit)	Characteristic TIME Scales (s)
Water	13 ± 10	74.5 ±
4.1	10 ± 4	103.6 ±
5.1	10 ± 2	98.5 ±
6.2	6 ± 2	169.9 ±
7.1	-	-
7.4	-	-
8.4	-	-

## Supplementary Materials

The following are available online at www.mdpi.com/1996-1944/10/2/216/s1.
