# Supplementary material for: Kinetic Analysis of the Uptake and Release of Fluorescein by Metal-Organic Framework Nanoparticles
Source: Materials (Basel). 2017 Feb 22;10(2):216. doi: 10.3390/ma10020216 (PMC5459203; doi:10.3390/ma10020216)
Supplement: Supplementary file 1 [file materials-10-00216-s001.pdf]

Supplementary Materials: Kinetic Analysis of the Uptake and Release of Fluorescein by Metal-Organic Framework Nanoparticles

Tobias Preiß, Andreas Zimpel, Stefan Wuttke and Joachim O. Rädler

Methods and Characterization

Powder X-ray diffraction (XRD) measurements were performed using a Bruker D8 diffractometer (Cu-$K_{\alpha 1}$ = 1.5406 Å; Cu-$K_{\alpha 2}$ = 1.5444 Å) in theta-theta geometry equipped with a Lynx-Eye detector. The powder samples were measured between 2° and 45° two theta, with a step-size of 0.05° two theta.

Nitrogen sorption measurements were performed on a Quantachrome Instruments Autosorb at 77 K. Sample outgassing was performed for 12 hours at 393 K. Pore size and pore volume were calculated by a NLDFT equilibrium model of N_2 on silica, based on the adsorption branch of the isotherms. BET surface area was calculated over the range of partial pressure between 0.05 – 0.20 p/p0. The pore volume was calculated based on the uptake (cm^3/g) at a relative pressure of 0.30 p/p0.

Dynamic light scattering (DLS) measurements were performed on a Malvern Zetasizer- Nano instrument equipped with a 4 mW He-Ne laser (633 nm) and an avalanche photodiode. The hydrodynamic radius of the particles was determined by dynamic light scattering in a diluted aqueous suspension.

Experimental section

Chemicals

Chromium(III) nitrate nonahydrate (99%, Aldrich, city, country), terephthalic acid (98%, Aldrich), ethanol (99%, Aldrich) Iron (III) chloride hexahydrate (Grüssing GmbH), trimesic acid (BTC, Aldrich).

Synthesis of MIL-101(Cr) Nanoparticles

The microwave synthesis of MIL-101(Cr) nanoparticles was based on a modified procedure reported in the literature. An amount of 20 mL (1.11 mol) of H_2O was added to 615 mg (3.70 mmol) terephthalic acid and 1.48 g $Cr(NO_3)_3 \cdot 9\ H_2O$ (3.70 mmol) [1]. This mixture was put into a Teflon tube, sealed and placed in the microwave reactor (Microwave, Synthos, Anton Paar). Four tubes were filled and inserted into the reactor: one tube contained the reaction mixtures described above; the remaining tubes including the reference tube with the pressure/temperature sensor (PT sensor) were filled with 20 mL H_2O. For the synthesis, a temperature programme was applied with a ramp of 4 min to 180 °C and a holding time of 2 min at 180 °C. After the sample had cooled down to room temperature, it was filtrated and washed with 50 ml EtOH to remove residual e.g., terephthalic acid. For purification, the filtrate was centrifuged and redispersed in 50 ml EtOH three times. The sample was centrifuged at 20000 rpm (47808 rcf) for 60 min.

Synthesis of MIL-100(Fe) Nanoparticles

For the microwave synthesis of MIL-100 (Fe) nanoparticles, iron(III) chloride hexahydrate (2.43 g, 9.00 mmol) and trimesic acid (0.84 g, 4.00 mmol) in 30 mL H_2O were put into a Teflon tube, sealed and placed in the microwave reactor (Microwave, Synthos, Anton Paar). The mixture was heated to 130 °C under solvothermal conditions (p = 2.5 bar) within 30 seconds, kept at 130 °C for 4 min and 30 s and the tube was cooled down to room temperature [1]. For the purification of the solid, the reaction mixture was centrifuged (20,000 rpm = 47,808 rcf, 20 min), the solvent was removed and the pellet was redispersed in 50 mL EtOH. This cycle was repeated two times and the dispersed solid was allowed to sediment overnight. The supernatant of the sedimented suspension was filtrated (filter discs grade: 391, Sartorius Stedim Biotech) three times, yielding MIL-100(Fe) nanoparticles.

Calculations

Particle Density

To calculate the number of particles per mg we assume spherical particles (Volume $V = \frac{4}{3}\,\pi\, r^3$, with NP Radius r) The mass of one NP m_{NP} is then m_{NP} = V × ϱ with the mass density ϱ of the NP material. The number of particles N in 1 mg is then $N = \frac{1\text{mg}}{m_{NP}}$. For MIL-100(Fe) nanoparticles we used a mean radius of $r_{MIL-100}$ =26.5 nm (obtained from TEM analysis) and a mass density of ρ_{MIL100} = 0.98 g/mL [2]. As a result we arrive at a mean mass per particle of m_{MIL100} = 0.076 fg and thus the number of N_{MIL100} = 1.31 × 10^{13} particles per milligramm of material. This corresponds to n_{MIL100} = 21.7 pmol. Respectively for MIL-101(Cr) nanoparticles with r_{MIL101} =9.45 nm and ρ_{MIL101} = 0.62 g/mL [3] we derived a mean particle mass of m_{MIL101} = 2.2 ag and thus N_{MIL101} =4.56 × 10^{14} particles per milligram (n_{MIL100} = 0.76 nmol).

On-Kinetics

To calculate the calculate the on-kinetics in a diffusion dominated process we adapt the theory of Adam and Delbrück [4]: The original formula for the mean time τ a molecule within a Volume with radius r needs to hit a sphere with radius R is $\tau = \frac{\left(1-\frac{r}{R}\right)^2}{3\,r\,D} R^3$ with D, the diffusion coefficient of the diffusing molecules. This function was adapted to the system at hand: for r we used the particles radius we derived from DLS measurements at used pH 5.1 of 400 nm, the diffusion coefficient for fluorescein was found to be 390 µm²/s (FCS measurement), the radius R of the volume was determined by calculating the mean solution volume per particle from the overall Volume V and the containing number of nanoparticles N: $R^3 = \frac{V}{N} = \frac{M}{c\,N_A}$. Where M is the molar mass of the nanoparticle, c the mass concentration and the Avogadro constant N_A. The molar Mass is derived by the volume of a sphere with the radius of one particle, its mass density ϱ and the Avogadro constant: $M = \frac{4}{3}\pi\, r^3\, \rho\, N_A$. This results in the following formula:

$$\tau(c_{NP}) = \frac{A \cdot 4\pi r^2 \rho}{72 c_{NP} D}\left(1 - \frac{2}{\sqrt[3]{\frac{4\pi\rho}{c_{NP}}}}\right)^2 + \tau_0 \approx \frac{A \cdot \pi r^2 \rho}{18\, c_{NP} D} + \tau_0$$

Whereas an offset τ_0 was added to compensate for the internal diffusion through the lattice and sorbtion that is represented in **Error! Reference source not found.** by the sum of τ_{intra} and τ_{on}. Here we used the mass density ϱ = 2 mg/cm³ that respects the filling of the nanoparticles with water (mass density of empty MOFs: 0.98mg/cm³ [2] + pore volume 1.030cm³/g [5] filled with water at 0.997mg/cm³ results in 2 mg/mL).

Supplementary Figure

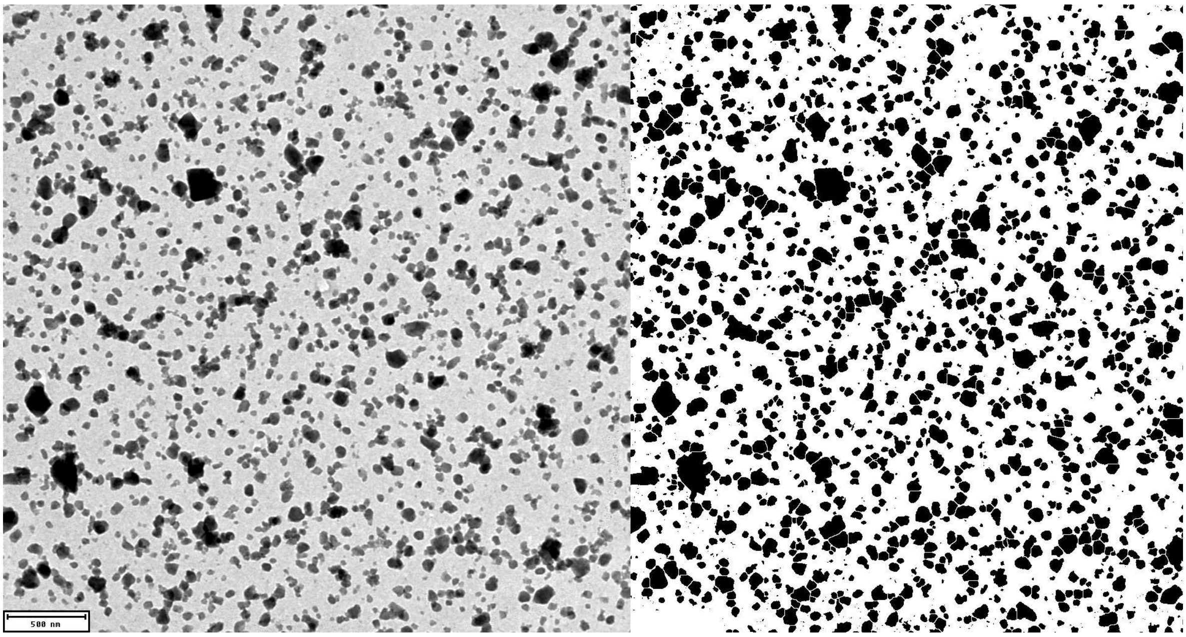

(**a**) MIL-100(Fe)

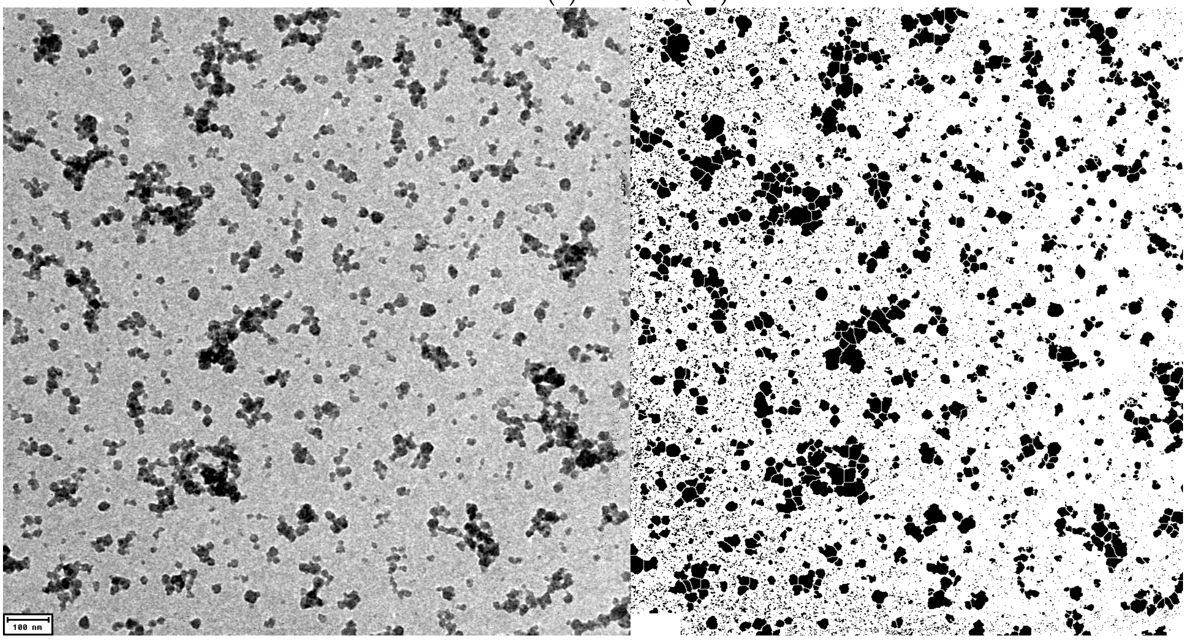

(**b**) MIL-101(Cr)

Figure S1. Juxtaposition of exemplary original TEM image (left) and processed image used for particle analysis (right) of (**a**) MIL-100(Fe) and (**b**) MIL-101(Cr). Original TEM image was converted to binary image. By applying watershed filter, NP that are close together are separated by a thin white line for the subsequent particle analysis. The "analyse particles" function of ImageJ was used to determine the area of all particles larger than 5 nm² (to get rid of background sparkles).

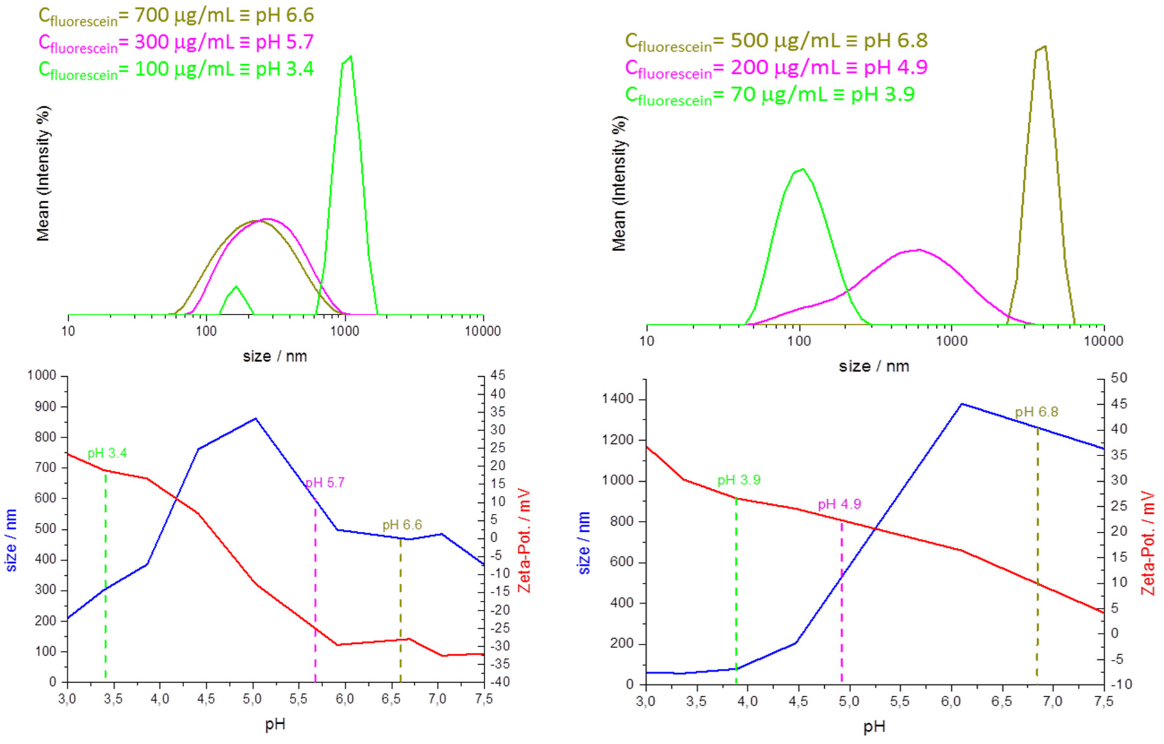

(**a**) MIL-101(Cr) (**b**) MIL-100(Fe)

Figure S2. DLS (blue) and Zeta-Potential (red) measurements of (**a**) MIL-100(Fe) (left) and (**b**) MIL-101(Cr) (right) at different pH in water.

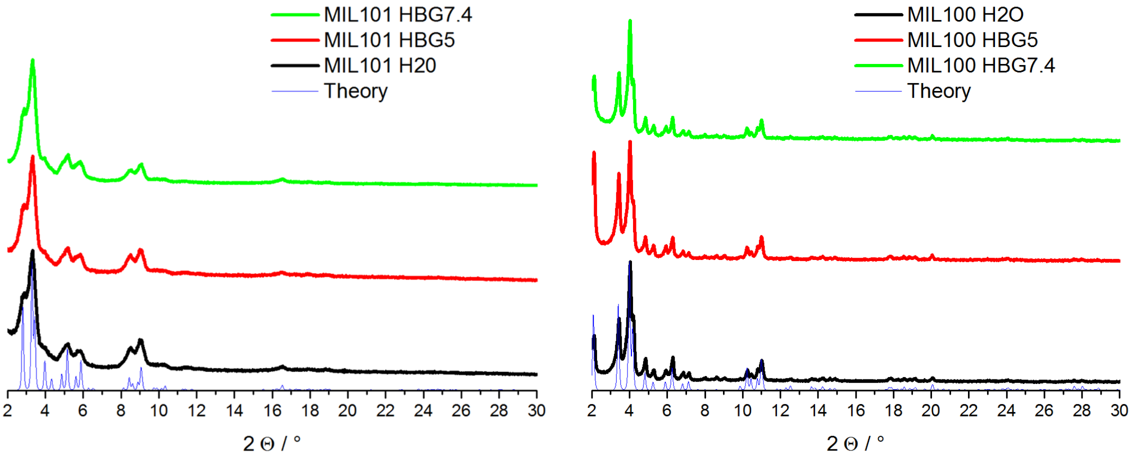

(**a**) (**b**)

Figure S3. XRD measurements of NPs before (black) and after incubation in buffer (red: in HBG pH = 5.0, green: in HBG pH = 7.4) certifies crystallinity and stability of MOF structure. Theoretical data is shown in blue.

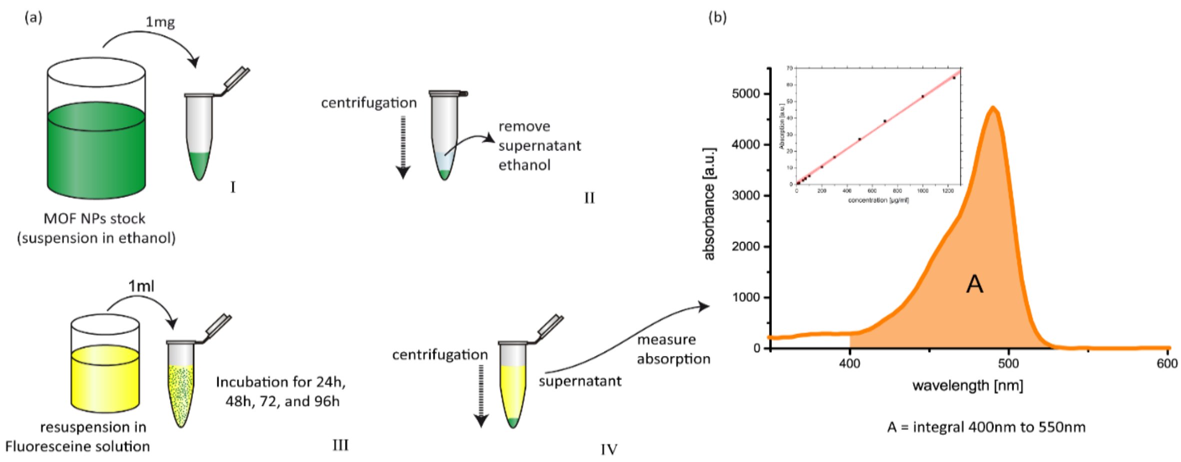

Figure S4. Assay for measuring the Payload capacity established, using UV/VIS absorption. Fluorescein solutions were used as calibration standard. (**a**) Preparation and loading process: 1 mg MOF NPs (I) were separated from ethanol (II), resuspended in fluorescein solutions of different concentrations and incubated for a certain time (III). For UV/VIS measurement the MOF NPs were separated from incubation solution (IV). (**b**) The absorption spectra of the supernatant solution as well as the original fluorescein solution were measured and integrated within the limits of 400 nm to 550 nm to determine the remaining amount of fluorescein in the supernatant. Inset: Fluorescein calibration curve with linear fit.

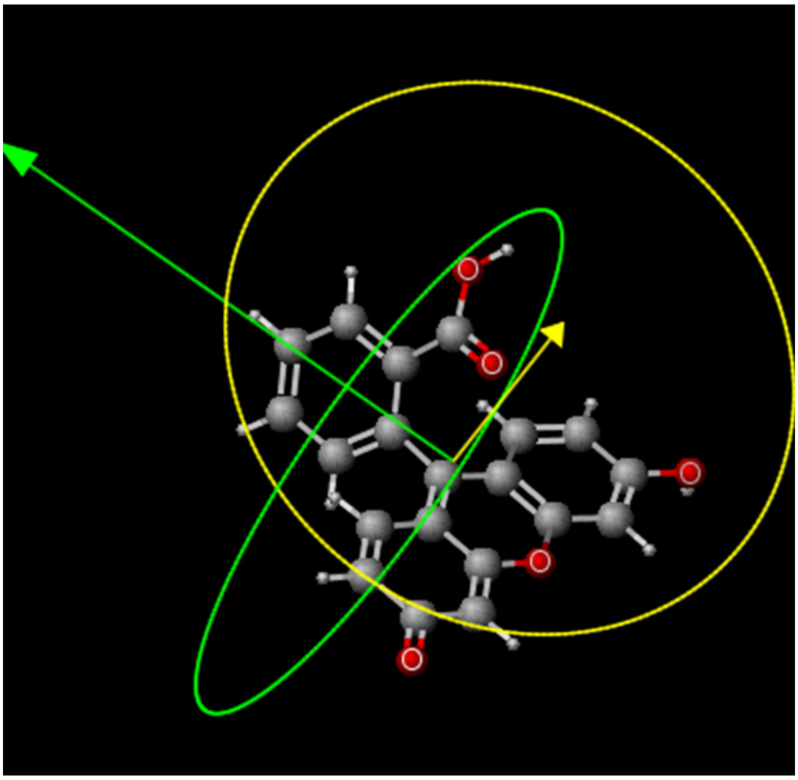

Figure S5. Structure of fluorescein molecule and its minimal and maximal projection area (MarvinSketch) max: radius = 6.27 Å; min: radius = 6.15 Å. Arrows indicate surface normal.

Release kinetics in HBG Buffer:

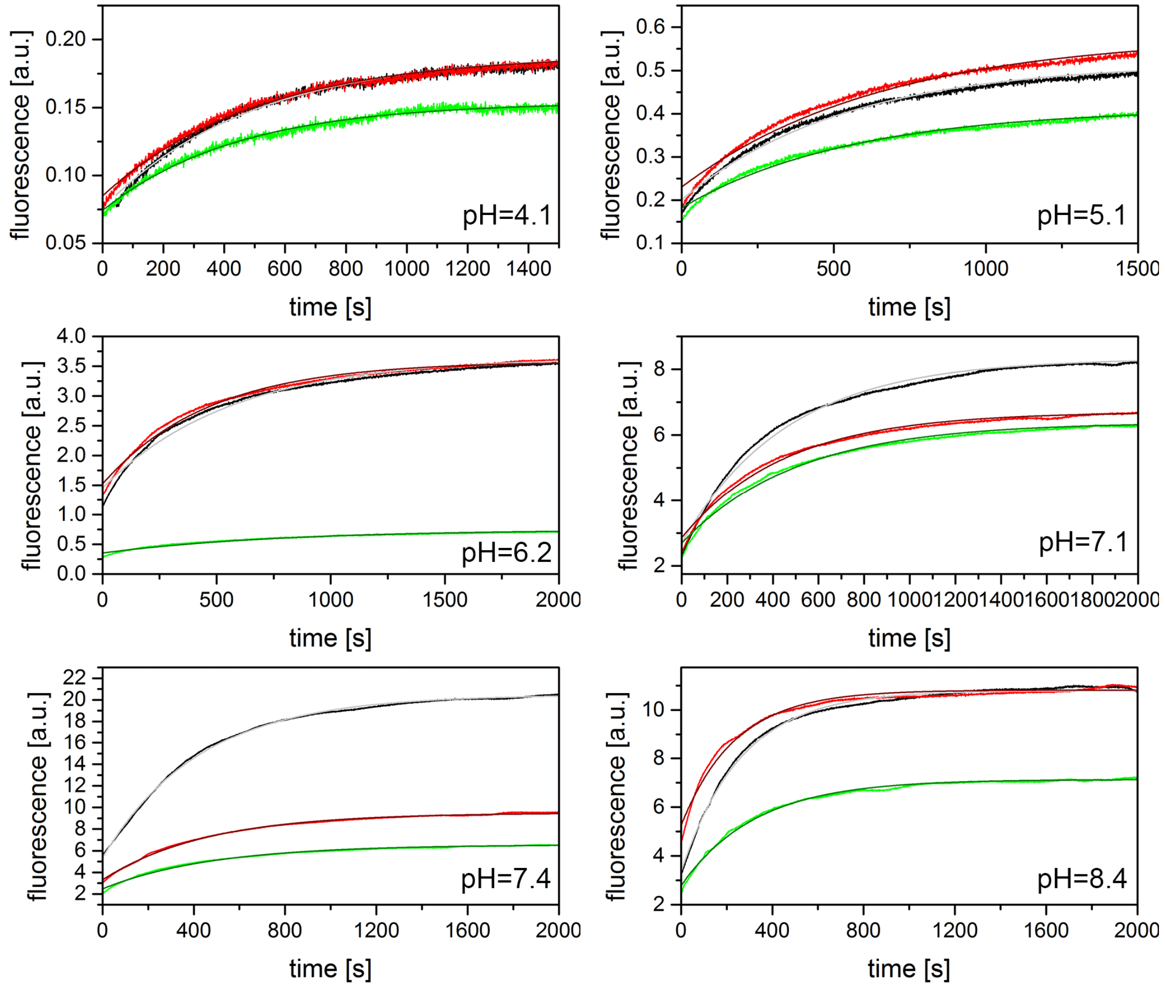

Figure S6. Release kinetics of fluorescein in MIL-100(Fe) NPs in HBG at depicted pH. Experiments were carried out in triplicate (black, red, green)

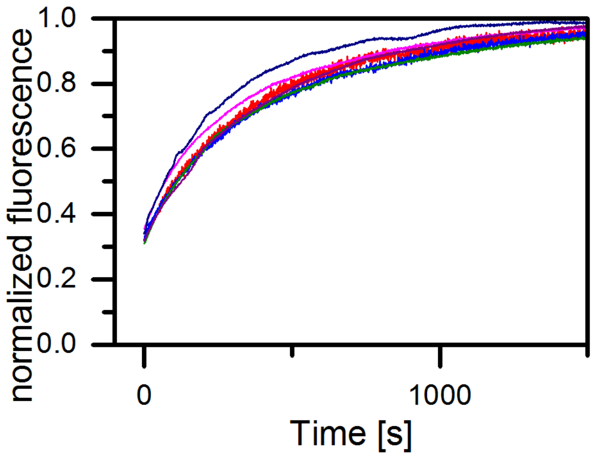

Figure S7. Release kinetic measurements at different pH values normalized to its final signal. pH = 4.1 (red); pH = 5.1 (blue); pH = 6.2 (green), pH = 7.1(pink) pH = 7.4 (violet), pH = 8.4 (dark blue)

Loading Kinetics

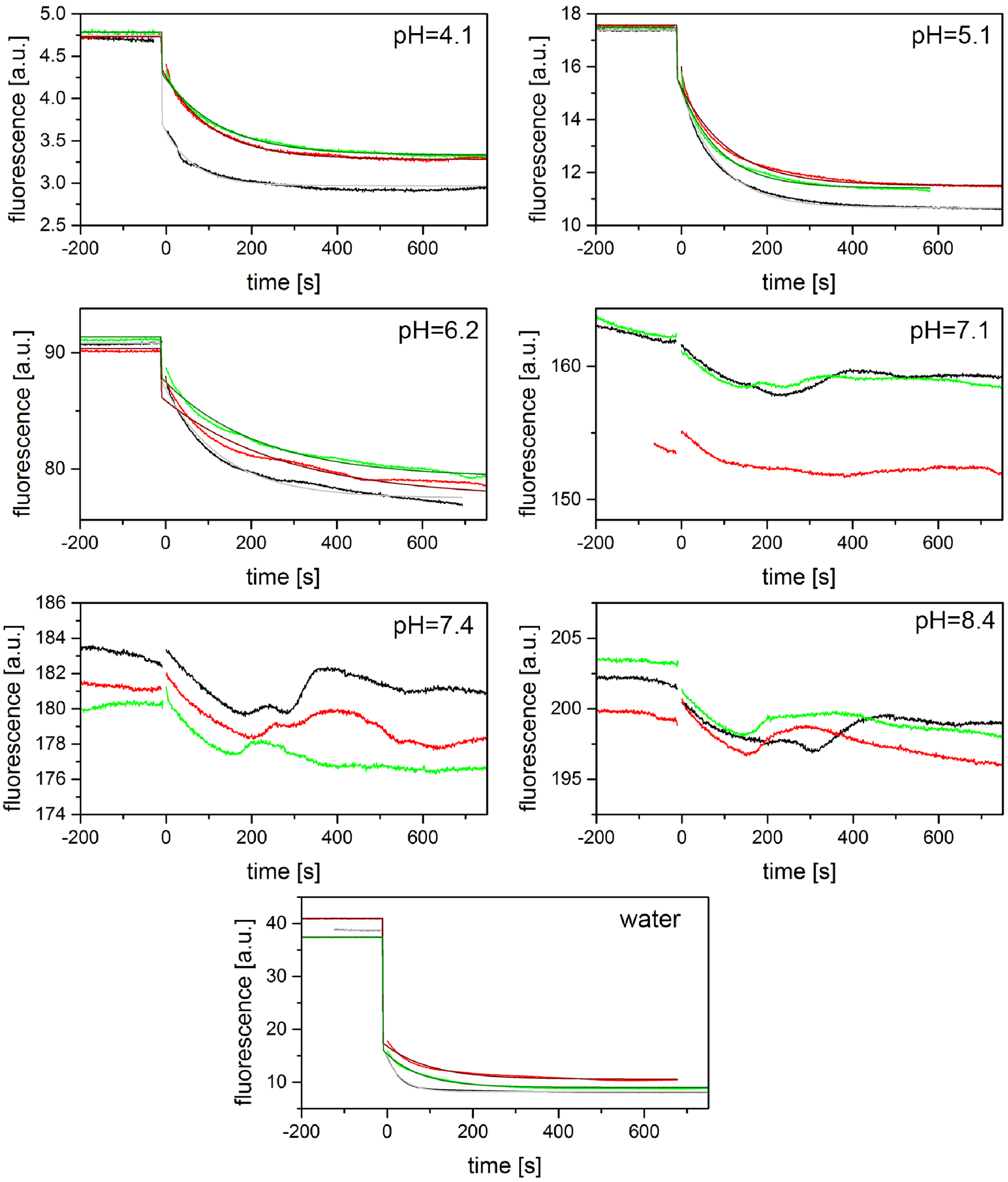

Figure S8. Loading kinetics of fluorescein into MIL-100(Fe) NPs in HBG at depicted pH and in water. Experiments were carried out in triplicate (black, red and green)

References

1. Wuttke, S.; Braig, S.; Preiß, T.; Zimpel, A.; Sicklinger, J.; Bellomo, C.; Rädler, J. O.; Vollmar, A. M.; Bein, T. MOF Nanoparticles Coated by Lipid Bilayers and Their Uptake by Cancer Cells. *Chem. Commun.* **2015**, *51*, 15752–15755.
2. Horcajada, P.; Surblé, S.; Serre, C.; Hong, D.-Y.; Seo, Y.-K.; Chang, J.-S.; Grenèche, J.-M.; Margiolaki, I.; Férey, G. Synthesis and Catalytic Properties of MIL-100(Fe), an iron(III) Carboxylate with Large Pores. *Chem. Commun.* **2007**, *100*, 2820–2822.
3. Férey, G.; Mellot-Draznieks, C.; Serre, C.; Millange, F.; Dutour, J.; Surblé, S.; Margiolaki, I. A Chromium Terephthalate-Based Solid with Unusually Large Pore Volumes and Surface Area. *Science* **2005**, *309*, 2040–

2042.

4. Adam, G.; Delbrueck, M. Reduction of Dimensionality in Biological Diffusive Processes. In *Structural Chemistry and Molecular Biology*; Rich, A.; Davidson, N., Eds.; Freeman: San Francisco, CA, USA, 1968; pp. 198–215.
5. Bernini, M. C.; Fairen-Jimenez, D.; Pasinetti, M.; Ramirez-Pastor, A. J.; Snurr, R. Q. Screening of Bio-Compatible Metal-Organic Frameworks as Potential Drug Carriers Using Monte Carlo Simulations. *J. Mater. Chem. B* **2014**, *2*, 766–774.
